# Pitfalls of the Semi-Quantitative Analyzing ^99m^Tc-Pyrophosphate Planar Images for Diagnosing Transthyretin Cardiac Amyloidosis: A Possible Solution

**DOI:** 10.3390/diagnostics12010094

**Published:** 2022-01-01

**Authors:** Yuankai Zhu, Ruping Pan, Dan Peng, Qingjian Dong, Xiaohua Zhu

**Affiliations:** Department of Nuclear Medicine, Tongji Hospital, Tongji Medical College, Huazhong University of Science and Technology, Wuhan 430030, China; ykzhu@hust.edu.cn (Y.Z.); panruping1109@hotmail.com (R.P.); danpeng715@163.com (D.P.); aiyi_aiyi@163.com (Q.D.)

**Keywords:** ^99m^Tc-pyrophosphate scintigraphy, cardiac amyloidosis, planar image, image analysis

## Abstract

Background: Two different approaches, 1-h heart-to-contralateral (H/CL) ratio and 3-h visual grading scale relative to ribs (VGSr), have been established to interpret ^99m^Tc-PYP planar images for the detection of amyloid transthyretin cardiac amyloidosis (ATTR-CA). Since they are prone to pitfalls, this pilot study aimed to explore the diagnostic practicality of the 3-h visual grading scale relative to the upper segment of sternum (VGSs) approach for interpreting ^99m^Tc-PYP planar images. Methods: A total of 42 patients were enrolled in this retrospective study. SPECT/CT approach and planar approaches including H/CL ratio, VGSr, and VGSs were utilized to interpret the ^99m^Tc-PYP images obtained at both 1 and 3 h. The classification criteria of the latest expert consensus recommendations were considered as the gold standard. The concordance between the interpretation of each approach and the gold standard was investigated. Results: In addition to 1- and 3-h SPECT/CT approaches, the interpretation of planar images using the 3-h VGSs approach was also applicable, which turns identical to the gold standard (κ = 1.000; *p* < 0.001). Conclusions: For the interpretation of ^99m^Tc-PYP planar images, the 3-h VGSs approach should be the optimal method, particularly in the case without available or feasible tomography imaging. Only one imaging session (planar and SPECT/CT) at 3 h would be sufficient for the detection of ATTR-CA, and favorable for patient satisfaction.

## 1. Introduction

Cardiac amyloidosis (CA) is a sort of infiltrative/restrictive cardiomyopathy, leading to significant mortality [1]. The most prevalent types of CA are amyloid transthyretin (ATTR) and amyloid light-chain (AL)-related, and the former is gradually considered as an underdiagnosed reason for heart failure [2,3]. Despite the initiation of novel targeted therapies, outcomes for advanced ATTR-CA remain bleak. Thus, reliable and early identification of affected patients would be crucial for further effective and timely treatment [4,5,6].

Invasive endomyocardial biopsy, as a traditional gold standard, can be avoided in subjects with suspected ATTR-CA, when ^99m^Tc-pyrophosphate (^99m^Tc-PYP) myocardial uptake is confirmed in the absence of immunoglobulin light chain abnormality [7,8]. According to the newly proposed expert consensus recommendations, even if diffuse ^99m^Tc-PYP uptake in the myocardium is visually verified on images of single-photon emission computed tomography (SPECT), further semi-quantitative analysis for planar images are still required [8]. Two different planar approaches, 1-h heart-to-contralateral (H/CL) ratio and 3-h visual grading scale relative to ribs (VGSr), have been established for interpreting ^99m^Tc-PYP planar images to distinguish AL-CA from ATTR-CA [8,9,10]. However, they are prone to pitfalls, as evaluation of planar images can be affected by radioactivity from overlying structures. Without evidence of ^99m^Tc-PYP myocardial uptake on SPECT images, both blood pool activity of left ventricular (LV) cavity and intense radiotracer uptake in adjacent ribs may increase the possibility of “false positive” interpretations of planar images [11]. Lack of congruence was reported between planar and SPECT imaging, and the correlation between H/CL ratio and VGSr approaches was even poorer [11,12]. Consequently, the existing protocols for interpreting planar images have been questioned.

Accordingly, the myocardial ^99m^Tc-PYP imaging protocol is constantly ameliorated by different centers, variably performed through planar scans and/or SPECT(/CT) at 1 and/or 3 h [11,13,14,15,16,17,18,19]. Compared with 1-h images, 3-h images have the merit of better radiotracer clearance from the LV cavity, which allows a more reliable interpretation of ^99m^Tc-PYP images. Besides, based on the clinical practice in our center, the 3-h visual grading scale relative to the upper segment of sternum (VGSs) approach was supposed to be superior to the canonical approaches for interpreting planar images. Using our proposed 3-h VGSs approach, the radiotracer activity in the mediastinum blood pool (overlapped with sternum) and LV cavity (overlapped with myocardium) should theoretically be offset, facilitating the assessment of actual relative radiotracer uptake in the myocardium to the bone. Thus, this pilot study aimed to verify the diagnostic practicality of the 3-h VGSs approach for interpreting ^99m^Tc-PYP planar images.

## 2. Materials and Methods

### 2.1. Patients

Fifty-one patients with suspected ATTR-CA referred for ^99m^Tc-PYP scintigraphy from March 2019 to June 2021 were retrospectively investigated. As 9 of them did not undergo SPECT/CT imaging at either 1- or 3-h time point, they were excluded from the final analysis. Demographics and clinical characteristics were reviewed via electronic medical record systems. Immunoglobulin light chain test was performed in 25 (59.5%) patients, while endomyocardial biopsy in 2 (4.8%) patients. Two patients with ATTR-CA underwent sequencing of the TTR gene. The study design was approved by the Institutional Review Board of Tongji Hospital, Tongji Medical College, Huazhong University of Science and Technology (Approval No. TJ-IRB20210713). The claim for informed consent was waived.

### 2.2. Image Acquisition

The procedure of ^99m^Tc-PYP scintigraphy was in accordance with the recent expert consensus recommendation [8]. Briefly, chest planar imaging followed by non-gated SPECT/CT imaging was performed 1 and 3 h after injecting 740MBq (20mCi) ^99m^Tc-PYP, on a dual-headed SPECT/CT camera (Discovery NM/CT 670, GE Medical Systems Israel, Functional Imaging). Low energy high-resolution collimator was used with an energy window of 15% centered at 140 keV. Duration for the anterior planar imaging was based on the acquisition of 750 K counts. For SPECT/CT imaging, the number of views for each detector was 32, and the time per stop was 20 s. The applied matrixes for planar and SPECT images were 256 × 256 and 128 × 128, respectively. Besides attenuation correction, low-dose CT was also utilized to confirm the localization of the myocardium when needed for interpreting SPECT/CT images [20,21].

### 2.3. Image Analysis

Both SPECT/CT and planar images were evaluated by two nuclear medicine experts respectively. Visualization of either diffuse or focal radiotracer uptake in the myocardium on SPECT/CT images was considered to be a positive SPECT/CT scan [12]. H/CL ratio and other two types of semi-quantitative visual grading scale (VGS) approaches were employed to interpret the 1- and 3-h planar images.

#### 2.3.1. H/CL Ratio 

**Figure 1:**
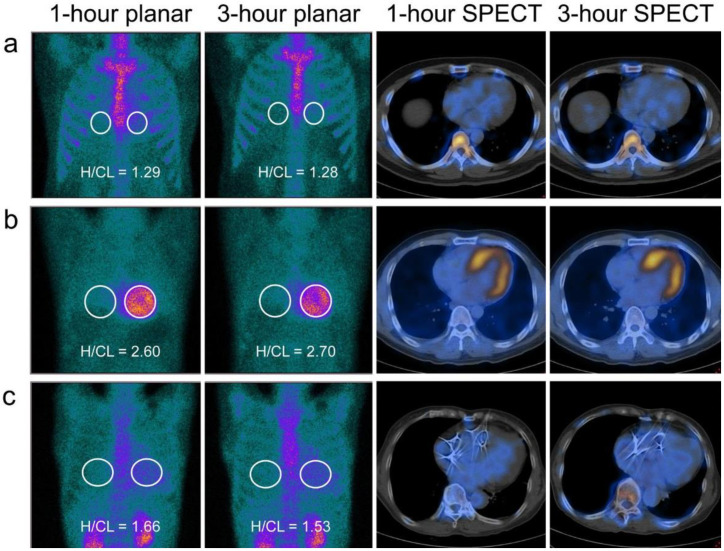
^99m^Tc-PYP planar and SPECT/CT scans obtained at 1 and 3 h in typical cases. White ellipse /circle of region of interest (ROI) was drawn over the precordium on anterior planar images, and mirrored to the contralateral chest for correcting the uptake in ribs and lung background. (**a**) The patient had negative planar studies at both 1 and 3 h based upon semi-quantitative visual grading scale relative to ribs (VGSr), visual grading scale relative to the upper segment of the sternum (VGSs), and heart-to-contralateral (H/CL) ratio approaches, respectively. 1- or 3-h SPECT/CT imaging allows identification of radiotracer activity in the left ventricular cavity other than the myocardium. (**b**) Despite the lack of heart failure symptoms, this patient had positive planar studies at both 1 and 3 h based upon semi-quantitative VGSr (both Grade 3), VGSs (both Grade 3) and H/CL ratio (2.60 and 2.70, respectively) approaches. Both 1- and 3-h SPECT/CT images showed diffuse ^99m^Tc-PYP uptake in the myocardium. As the immunoglobulin light chain test was negative and Asp38Asn mutation was detected, a definite diagnosis of hereditary ATTR-CA was achieved without processing an invasive endomyocardial biopsy. (**c**) Based upon semi-quantitative VGSr (both Grade 3) and H/CL ratio (1.66 and 1.53, respectively) approaches at 1 and 3 h, as well as 1-h VGSs (Grade 2) approach, the patient was considered as having positive planar studies. However, this “false positive” interpretation was refuted by Grade 1 referring to 3-h VGSs, with concordant to no radiotracer myocardial uptake on 1- and 3- hour SPECT/CT images.

An elliptical/circular region of interest (ROI) was drawn over the precordium on anterior planar images, and mirrored to the contralateral chest for correcting the uptake in ribs and lung background (Figure 1). The ROI should be positioned to minimize overlap with sternal or focal ribs and to maximize the coverage of the heart by avoiding adjacent lungs. The ratio of precordium ROI to contralateral chest ROI (mean counts per pixel) was calculated as the H/CL ratio. A H/CL ratio ≥1.5 on a 1-h image or ≥1.3 on a 3-h image was considered positive [11,22,23,24].

#### 2.3.2. VGS

Semi-quantitative VGS approach, comparing the radiotracer uptake in myocardium relatively to ribs (VGSr) or the upper segment of the sternum (VGSs), was performed on anterior planar images as previously described [8,9]. Specifically, absent ^99m^Tc-PYP myocardial uptake was considered as Grade 0, myocardial uptake less than ribs (or upper segment of the sternum) as Grade 1, myocardial uptake equal to ribs (or upper segment of the sternum) as Grade 2, and myocardial uptake greater than ribs (or upper segment of the sternum) as Grade 3, respectively. For the interpretation of planar images using the VGSr or VGSs approach, Grade ≥2 was considered positive.

The classification criteria of recent expert consensus recommendations were referred to as the gold standard for the purpose of the present study [8]. The first step was evaluating planar and SPECT/CT images to confirm diffuse myocardial tracer uptake. Subjects without myocardial uptake of ^99m^Tc-PYP on the SPECT/CT images were classified as “non-suggestive of ATTR-CA”. If ^99m^Tc-PYP myocardial uptake was visually verified on the SPECT/CT images, subjects with Grade ≥2 on planar images using 3-h VGSr approaches were classified as “strongly suggestive of ATTR-CA”. Despite ^99m^Tc-PYP myocardial uptake confirmed on the SPECT/CT images, subjects with either 1-h H/CL ratio of 1–1.5 or Grade 1 referring to 3-h VGSr approaches were classified as “equivocal of ATTR-CA”. Patients who tested negative in the immunoglobulin light chain test with “strongly suggestive of ATTR-CA” in ^99m^Tc-PYP imaging, could be definitely diagnosed as ATTR-CA without processing an invasive endomyocardial biopsy. Both ATTR-CA and AL-CA can be excluded, if the immunoglobulin light chain test was negative with Grade 0 ^99m^Tc-PYP uptake in the myocardium.

### 2.4. Statistical Analysis

All data were analyzed using SPSS software (version 25.0; IBM SPSS Statistics). Continuous variables were reported as mean (SD) or median (interquartile range [IQR]) values as appropriate, while categorical variables were as frequency (percentage). Group differences were tested using the two-sample *t*-test, Mann–Whitney test, or Fisher exact test as appropriate. Concordance between classification results of every single approach and classification criteria of expert consensus recommendations was performed using Cohen’ κ-coefficient. *p* values < 0.05 were considered statistically significant.

## 3. Results

Forty-two patients (15 females; mean age ± SD, 59.1 ± 13.8 years) were finally included in this pilot study. Among them, 5 patients (11.9%) were classified as “strongly suggestive of ATTR-CA”, while the other 37 patients (88.1%) as “non-suggestive of ATTR-CA”, based on the classification criteria of expert consensus recommendations [8]. Nevertheless, no subjects were classified as “equivocal of ATTR-CA” in this study, since all patients with positive SPECT/CT scan were classified as Grade ≥ 2 on planar images using the 3-h VGSr approach. Six patients with positive findings in immunoglobulin light chain test were all classified as “non-suggestive of ATTR-CA”. As all the 5 patients with “strongly suggestive of ATTR-CA” tested negative in the immunoglobulin light chain test, they were definitely diagnosed with ATTR-CA without processing an invasive endomyocardial biopsy. Among them, two brothers were further diagnosed as hereditary ATTR-CA, due to the detection of Asp38Asn mutation in genetic testing.

The demographic and clinical characteristics of the subjects were summarized in Table 1. Valvular/annular calcification was found only in 1 patient, but without significantly increased uptake of ^99m^Tc-PYP compared with the surrounding blood pool. Though 8 patients had a history of rib fracture, all these lesions were not located at the precordial region. The thickness of both interventricular septum and LV posterior wall of the subjects with “strongly suggestive of ATTR-CA”, were significantly greater than those with “non-suggestive of ATTR-CA” (*p* < 0.001 and = 0.004, respectively). Besides, patients with “strongly suggestive of ATTR-CA” showed a significantly greater estimated glomerular filtration rate (eGFR) and a higher level of Troponin I (*p* = 0.033 and 0.037, respectively). The H/CL ratio, VGSr and VGSs at both 1 h (*p* < 0.001, =0.013 and <0.001, respectively) and 3 h (*p* < 0.001 in each comparison), were significantly higher in patients with “strongly suggestive of ATTR-CA”, when compared to those with “non-suggestive of ATTR-CA”.

The classification results of H/CL ratio, VGSr, VGSs and SPECT/CT approaches at both 1 h (κ = 0.806, *p* < 0.001; κ = 0.151, *p* = 0.092; κ = 0.463, *p* = 0.002 and κ = 1.000, *p* < 0.001; respectively) and 3 h (κ = 0.604, 0.552, 1.000 and 1.000, respectively; *p* < 0.001 in each comparison), compared to the classification criteria of expert consensus recommendations, were presented in a contingency table (Table 2). In addition to 1- and 3-h SPECT/CT approaches, the interpretation of planar images using the 3-h VGSs approach was also identical to the classification criteria. Six patients with “false positive” findings according to the 3-h VGSr approach showed no evidence of radiotracer myocardial uptake in their corresponding SPECT/CT images. The same also happened in 2, 5, 23, and 8 patients by using 1-h H/CL ratio, 3-h H/CL ratio, 1-h VGSr, and 1-h VGSs approaches, respectively. Among these “false positive” patients, only radiotracer activity in the LV cavity was observed in SPECT/CT images. Representative images were displayed in Figure 1.

## 4. Discussion

We retrospectively evaluated 42 patients investigated for ATTR-CA undergoing ^99m^Tc-PYP scintigraphy. Five patients were classified as “strongly suggestive of ATTR-CA”, and the others as “non-suggestive of ATTR-CA”, according to the classification criteria of recent expert consensus recommendations [8]. In addition to 1- and 3-h SPECT/CT approaches, the interpretation of planar images by referring to the 3-h VGSs approach was also applicable, which turns identical to the classification criteria.

The ^99m^Tc-PYP SPECT imaging were applied to verify myocardial tracer uptake, rather than radioactivity in the LV cavity or overlapped bones, which often lead to a “false positive” interpretation of planar images [19]. According to the newly proposed expert consensus recommendations, even if diffuse ^99m^Tc-PYP uptake in the myocardium is visually verified by SPECT images, further semi-quantitative analysis on planar images are still required [8]. In the present study, neither 1- nor 3-h ^99m^Tc-PYP SPECT/CT images combined with subsequent semi-quantitative interpretation of planar images led to an “equivocal of ATTR-CA”. Therefore, single SPECT/CT could independently be an alternate classification criterion for ^99m^Tc-PYP imaging [12,13,19]. Furthermore, patients with early-stage of ATTR-CA may present local patchy radiotracer uptake in the myocardium, which further underlines the importance of SPECT/CT imaging to identify the radiotracer distribution in a non-diffuse pattern [25]. The newly developed SPECT/CT system equipped with high-sensitivity 360-degree Cadmium Zinc Telluride cameras could not only reduce the imaging time but also improve diagnostic practicality by using absolute quantitation analysis [26].

For the present, a delayed 3-h SPECT/CT scan is recommended at least, when excessive activity in the LV cavity is noticed on 1-h SPECT/CT images [8]. Though radiotracer activity in the LV cavity is not always completely absent, particularly in patients with low cardiac output or renal failure, it might be diminished to some extent 3 h after injection. In common with our results, other studies also indicated that the uptake pattern of radiotracer in SPECT/CT was identical at 1 and 3 h [11,13]. More data are required to confirm that a single 1-h or 3-h SPECT/CT imaging could be sufficient to achieve identical diagnostic efficacy.

With SPECT imaging alone, however, distinguishing the uptake of myocardium from LV cavity remains imprecise [17]. The SPECT/CT system provides attenuation correction for SPECT images, improving the recognition of myocardial boundaries, but with additional radiation exposure. In contrast, planar imaging has a short imaging time and low dose, which is especially suitable for patients with heart failure who are unable to lie flat for a long duration in SPECT/CT imaging. Accordingly, 1-h H/CL ratio and 3-h VGSr, have been used to semi-quantitatively analyze the ^99m^Tc-PYP planar images [8,9,10]. However, the evaluation of planar images can be affected by radioactivity from overlapped structures such as the LV cavity and adjacent ribs, which may cause an overestimation of ^99m^Tc-PYP uptake in the myocardium [11]. The optimal method of drawing ROI for H/CL ratio calculation is not yet unequivocally specified. Unreasonable H/CL value should be taken with caution, especially in patients with either massive right pleural effusion or excessive radiotracer activity in the cardiac ventricle extending to the right side of the sternum [27,28]. Consequently, it has been suggested that the interpretation of ^99m^Tc-PYP scintigraphy referring to H/CL ratio shows poor correlation with that according to either VGSr or SPECT approach, and should not be utilized in the diagnosis of ATTR-CA [12].

**Figure 2:**
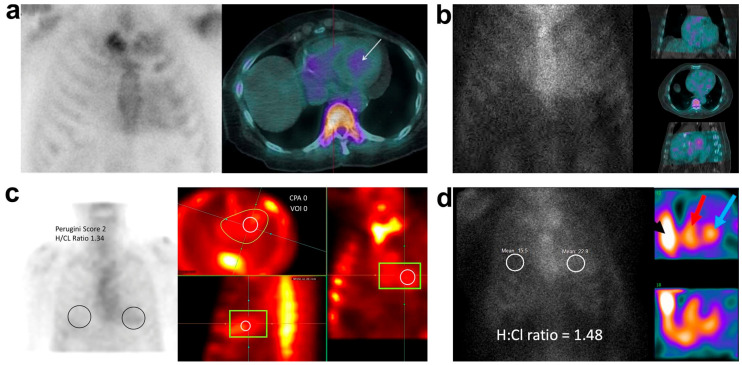
“False positive” interpretations on ^99m^Tc-PYP planar images. (**a**) A planar scan was performed at 1 h and interpreted as Grade 2 uptake using a visual grading scale relative to ribs (VGSr), with a heart-to-contralateral (H/CL) ratio of 1.5. However, ^99m^Tc-PYP scan at 3 h showed Grade 1 planar uptake (VGSr), with radiotracer within the left ventricular blood pool (white arrow) but no myocardial uptake on SPECT/CT images [7]. (**b**) A “positive” planar study at both 1 and 3 h was determined based upon Grade 2–3 uptake (VGSr) and H/CL ratio of 1.63 and 1.48, respectively. However, this “false positive” interpretation was refuted by Grade 1 uptake referring to visual grading scale relative to upper segment of sternum (VGSs) approach, with concordant to no radiotracer myocardial uptake on SPECT/CT images [11]. (**c**) In an AL-CA patient, planar imaging at 3 h was interpreted as “positive”, based upon Grade 2 uptake (VGSr) with a H/CL ratio of 1.34 (black circle). No radiotracer myocardial uptake (the range between green box/irregular circle and white circle) was found on SPECT images, and this “false positive” interpretation can be also refuted by using the VGSs approach [14]. (**d**) A “positive” planar study at 3 h was determined based upon Grade 2 uptake (VGSr) and H/CL ratio of 1.48 (white circle). Only blood pool activities of right (red arrow) and left (blue arrow) ventricles, and intense radiotracer uptake in the sternum (black arrow) were indicated on 3-h SPECT images [31]. Grade 1 uptake using the VGSs approach could reclassify this case as “false positive” as well. (All the reprint permissions have been obtained from the publishers).

Semi-quantitative VGSr was recommended to interpret ^99m^Tc-PYP planar images in accordance with the Perugini score system, which was derived from the interpretation of ^99m^Tc-3,3-diphosphono-1,2-propanodicarboxylic acid (^99m^Tc-DPD) planar images [8,9]. Though no direct comparison between ^99m^Tc-PYP and ^99m^Tc-DPD has been reported, the uptake degree of different radiotracers in the myocardium is indeed different [29]. A greater proportion of subjects with Grade 3 referring to VGSr, was found in centers utilizing ^99m^Tc-PYP (62.4%) compared to those in centers using ^99m^Tc-DPD (10.7%). In our present study, 6 patients with Grade ≥2 referring to 3-h VGSr did not present myocardial tracer uptake, but radiotracer activity in LV cavity on SPECT/CT images. Meanwhile, the 3-h VGSs approach, as well as 1- and 3-h SPECT/CT approaches, reclassified these “false positive” cases as being “negative”. Likewise, when excessive activity in the LV cavity was noticed on planar images, the consequent “false positive” interpretation of typical cases displayed in numerous studies (Table 3 and Figure 2), could be refuted if using our newly proposed 3-h VGSs approach without further verification by using tomography [7,11,12,14,27,28,30,31,32].

The upper segment of the sternum is mainly overlapped with the mediastinal blood pool on anterior planar images. Therefore, the radiotracer activity in the mediastinum blood pool and LV cavity should theoretically be offset using the VGSs approach, which is a potential advantage of calculating relative radiotracer uptake in the myocardium to the sternum. Compared with 1-h planar images, 3-h planar images are more favorable due to a better radiotracer clearance from the LV cavity, which allows an easier and more reliable interpretation of planar images. Thus, we suggest that the 3-h VGSs approach should be an optimal semi-quantitative grading method to estimate the ^99m^Tc-PYP myocardial uptake through planar images.

In the early stage of ATTR amyloidosis without cardiac involvement, there might be no significant uptake of ^99m^Tc-PYP in the myocardium through SPECT/CT imaging. Despite normal cardiac evaluation in these asymptomatic patients with ATTR gene mutations or extra-cardiac ATTR deposits, follow-up repeat ^99m^Tc-PYP scan allows early identification of the myocardial uptake conversion from negative to positive [33]. Likewise, one patient without heart failure symptoms in our study was referred for ^99m^Tc-PYP imaging after his brother was diagnosed with ATTR-CA. A definite diagnosis of ATTR-CA was ultimately established in this asymptomatic patient, by combining ^99m^Tc-PYP imaging (Figure 1b) and immunoglobulin light chain test. Therefore, cardiac ^99m^Tc-PYP imaging is recommended for screening subjects at high risk of ATTR-CA. In patients with advanced ATTR-CA, however, serial ^99m^Tc-PYP scanning failed to indicate significant changes in the degree of myocardial uptake, despite obvious clinical progression over a long-term follow-up [34].

The current study has several limitations. Firstly, considering the potential risk, endomyocardial biopsy, which is the traditional gold standard for CA diagnosis and is dependent on the expertise of a pathologist, was obtained in only 2 patients. Consistent with expert consensus recommendations, an endomyocardial biopsy can be avoided in subjects with suspected ATTR-CA if ^99m^Tc-PYP myocardial uptake is confirmed in the absence of immunoglobulin light chain abnormality [8,35]. Secondly, owing to specific referral patterns, the selection bias cannot be excluded in this retrospective study. A higher proportion of renal failure was found in patients with “non-suggestive of ATTR-CA”, which could result in prolonged radiotracer retention in the blood pool. Thirdly, since this is a pilot study for a relatively rare disease, merely limited cases were classified into each category. Despite these limitations, the prominent finding was the feasibility of the 3-h VGSs approach for the diagnosis of ATTR-CA, which could also be verified through re-evaluating some typical cases shown in other equivocal studies [7,11,12,14,27,28,30,31,32].

## 5. Conclusions

The newly proposed 3-h VGSs approach should be superior to canonical 1-h H/CL ratio or 3-h VGSr approaches for the interpretation of ^99m^Tc-PYP planar images. Compared to the dual-phase imaging with ^99m^Tc-PYP, 3-h imaging only (planar and SPECT/CT) could provide identical diagnostic information for the detection of ATTR-CA.

## Figures and Tables

**Table 1:** Demographic and clinical characteristics of patients referred for ^99m^Tc-PYP scintigraphy.

Characteristics	Diagnostic Criteria		*p*-Value
	Non-Suggestive (*n* = 37)	Strongly Suggestive (*n* = 5)	
Age, years	59.7 ± 14.0	54.6 ± 11.7	0.440
Female	15 (40.5%)	0 (0%)	0.142
CAD	10 (27.0%)	2 (40.0%)	0.613
Heart failure	30 (81.1%)	4 (80.0%)	1.000
Hypertension	16 (43.2%)	1 (20.0%)	0.632
Diabetes mellitus	10 (27.0%)	1 (20.0%)	1.000
Hyperlipidemia	12 (32.4%)	0 (0%)	0.298
Atrial fibrillation	12 (32.4%)	1 (20.0%)	1.000
Myocardial infarction (<4 weeks)	0 (0%)	0 (0%)	N/A
Rib fracture	8 (21.6%)	0 (0%)	0.564
Valvular/annular calcification	1 (2.7%)	0 (0%)	1.000
Hydroxychloroquine therapy	0 (0%)	0 (0%)	N/A
Disturbances in conduction	17 (45.9%)	3 (60.0%)	0.656
LV ejection fraction, %	55.0 (39.5–60.5)	44.0 (41.0–51.5)	0.286
Interventricular septum thickness on echo, cm	1.40 (1.10–1.60)	2.00 (1.85–2.45)	<0.001
Posterior wall thickness on echo, cm	1.20 (1.00–1.35)	1.60 (1.45–2.45)	0.004
Troponin I, ng/mL	40.4 (17.9–142.4)	231.0 (68.9–337.2)	0.037
BNP, pg/mL	1747.0 (614.0–6613.0)	4011.0 (914.5–5065.5)	0.763
Creatinine, mg/dL	93.0 (78.0–130.0)	72.0 (59.5–96.5)	0.061
eGFR, mL/min/1.73 m^2^	59.8 (45.0–86.3)	99.7 (77.2–108.4)	0.033
1-h H/CL ratio	1.21 (1.13–1.32)	1.99 (1.63–2.39)	<0.001
3-h H/CL ratio	1.22 (1.12–1.27)	2.00 (1.63–2.36)	<0.001
1-h VGSr	0/14/11/12	0/0/0/5	0.013
3-h VGSr	4/27/6/0	0/0/0/5	<0.001
1-h VGSs	0/29/8/0	0/0/2/3	<0.001
3-h VGSs	4/33/0/0	0/0/2/3	<0.001

Ordinal categorical variable data refer to the number of patients with Grade 0/ Grade 1/ Grade 2/ Grade 3; CAD, coronary artery disease; LV, left ventricular; BNP, brain natriuretic peptide; eGFR, estimated glomerular filtration rate; H/CL, heart-to-contralateral; VGSr, visual grading scale relative to ribs; VGSs, visual grading scale relative to the upper segment of the sternum.

**Table 2:** Comparison of classification results between diagnostic criteria and various interpretation approaches for ^99m^Tc-PYP scintigraphy.

Methods	Diagnostic Criteria		*p*-Value	*Kappa* (κ)
	Non-Suggestive (*n* = 37)	Strongly Suggestive (*n* = 5)		
1-h H/CL ratio	35/2	0/5	<0.001	0.806
3-h H/CL ratio	32/5	0/5	<0.001	0.604
1-h VGSr	14/23	0/5	0.092	0.151
3-h VGSr	31/6	0/5	0.001	0.552
1-h VGSs	29/8	0/5	0.002	0.463
3-h VGSs	37/0	0/5	<0.001	1.000
1-h SPECT	37/0	0/5	<0.001	1.000
3-h SPECT	37/0	0/5	<0.001	1.000

Data refer to the number of patients with negative versus positive; H/CL, heart-to-contralateral; VGSr, visual grading scale relative to ribs; VGSs, visual grading scale relative to the upper segment of the sternum.

**Table 3:** “False positive” interpretations on ^99m^Tc-PYP planar images were re-evaluated by using our proposed 3-h semi-quantitative grading (relative to the upper segment of the sternum) approach.

References	[Figure]^#^	H/CL (1-h)	H/CL (3-h)	VGRr (1-h)	VGRr (3-h)	VGRs (3-h)	SPECT (3-h)
Hanna, M. et al., [7]	[Figure 4]^#^	1.5	N/A	Grade 2	Grade 1	Grade 1	Negative
Sperry, B.W. et al., [11]	[Figure 2B]^#^	1.63	1.48	Grade 3	Grade 2	Grade 1	Negative
Asif, T. et al., [12]	[Figure 4]^#^	1.33	1.2	Grade 3	Grade 2	Grade 1	Negative
Miller, R.J.H. et al., [14]	[Figure 2]^#^	N/A	1.34	N/A	Grade 2	Grade 1	Negative
Tsuda, N. et al., [27]	[Figures A–D]^#^	N/A	N/A	N/A	Grade 2	Grade 1	Negative
Régis, C. et al., [28]	[Figure 2A]^#^	1.96	N/A	Grade 2	N/A	Grade 1 *	Negative *
Murray, C.S.G. et al., [30]	[Figures 3,4]^#^	1.53	N/A	Grade 3	N/A	Grade 1 *	Negative *
Poterucha, T.J. et al., [31]	[Figures 1–3]^#^	N/A	1.48	N/A	Grade 2	Grade 1	Negative
Butera, B. et al., [32]	[Figure 1]^#^	N/A	1.5	N/A	Grade 2	Grade 1	Negative

[Figure]^#^ refers to the figures in the corresponding references; * Only 1-h images available; H/CL, heart-to-contralateral; VGSr, visual grading scale relative to ribs; VGSs, visual grading scale relative to the upper segment of the sternum; A H/CL ratio ≥1.5 on a 1-h image or ≥1.3 on the 3-h image was considered positive; For the interpretation of planar images using VGSr or VGSs approach, Grade ≥2 was considered positive.

## Data Availability

The datasets generated during and/or analyzed during the current study are available from the corresponding author upon reasonable request.
